# Cut-Lengths of Perennial Ryegrass Leaf-Blades Influences In Vitro Fermentation by the Anaerobic Fungus *Neocallimastix frontalis*

**DOI:** 10.3390/microorganisms8111774

**Published:** 2020-11-11

**Authors:** Hugo R. Jimenez, Joan E. Edwards, Ruth Sanderson, Alison H. Kingston-Smith, Neil R. McEwan, Michael K. Theodorou

**Affiliations:** 1 Institute of Biological, Environmental and Rural Sciences, Aberystwyth University, Aberystwyth SY23 3EE, UK; hjimenez@agrosavia.co (H.R.J.); joanee2002@hotmail.com (J.E.E.); rts@aber.ac.uk (R.S.); n.mcewan@rgu.ac.uk (N.R.M.); 2 Corporación Colombiana de Investigación Agropecuaria-Agrosavia, Bogotá CI Tibaitatá-Km 14, Colombia; 3 School of Pharmacy and Life Sciences, Robert Gordon University, Aberdeen AB10 7GJ, UK; 4 Department of Agriculture and the Environment, Harper Adams University, Newport TF10 8NB, UK

**Keywords:** *Neocallimastigomycota*, fermentation kinetics, gas production, perennial ryegrass, *Neocallimastix frontalis*

## Abstract

Anaerobic fungi in the gut of domesticated and wild mammalian herbivores play a key role in the host’s ability to utilize plant biomass. Due to their highly effective ability to enzymatically degrade lignocellulose, anaerobic fungi are biotechnologically interesting. Numerous factors have been shown to affect the ability of anaerobic fungi to break down plant biomass. However, methods to reduce the non-productive lag time in batch cultures and the effect of leaf-blade cut-length and condition on the fungal fermentation are not known. Therefore, experimentation using a novel gas production approach with pre-grown, axenic cultures of *Neocallimastix frontalis* was performed using both fresh and air-dried perennial ryegrass leaf-blades of different cut-lengths. The methodology adopted removed the lag-phase and demonstrated the digestion of un-autoclaved leaf-blades. Fermentation of leaf-blades of 4.0 cm cut-length produced 18.4% more gas yet retained 11.2% more apparent DM relative to 0.5 cm cut-length leaf-blades. Drying did not affect fermentation by *N. frontalis*, although an interaction between drying and leaf-blade cut-length was noted. Removal of the lag phase and the use of un-autoclaved substrates are important when considering the biotechnological potential of anaerobic fungi. A hypothesis based upon sporulation at cut surfaces is proposed to describe the experimental results.

## 1. Introduction

Anaerobic fungi (*Neocallimastigomycota*) naturally occur in the gut of many mammalian herbivores, where they play a key role in the ability of the host to utilize dietary plant material [1,2]. Anaerobic fungi are one of the most potent fibre degrading microorganisms in the known biological world due to their potent fibrolytic enzymes combined with their ability to physically breakdown lignocellulosic plant material [3]. Due to their ability to form stable co-cultures with methanogenic archaea [4], the potential contribution of anaerobic fungi to methane production in anaerobic digestion (AD) has also been highlighted [5,6]. As such, anaerobic fungi have received much interest from the biotechnological perspective, particularly in terms of sustainable production of renewable energy and the production of platform chemicals from lignocellulosic feedstocks [7,8].

In the rumen, anaerobic fungi are considered to be primary colonisers of freshly ingested plant biomass [9]. Following zoospore location, attachment and encystment on plant fibres, anaerobic fungi develop an extensive rhizoidal network which penetrates deep into plant tissues [10,11,12]. It is the high specific activity of the fibrolytic enzymes associated with and/or released from fungal rhizoids that plays a key role in the disruption and enzymatic hydrolysis of plant structural carbohydrates. It has been shown that the carbohydrate active enzymes (CAZymes) of anaerobic fungi are assembled into cellulosomes [13]. These multi-modular structures enable the structured concentration and co-ordination of numerous classes of CAZyme that have different enzymatic properties [13].

Numerous factors have been shown to affect anaerobic fungal populations and/or their ability to breakdown plant material [1]. Co-cultivation with methanogens or other hydrogen-consuming bacteria stimulates anaerobic fungal cellulolysis [14]. Continuous-flow cultures of anaerobic fungi produce up to 30 times more cell-wall degrading enzymes than corresponding batch cultures [15]. The frequency of inoculum transfer has also been shown to have a significant impact on the productivity of anaerobic fungus and methanogen enrichment co-cultures [16].

It has been previously suggested that anaerobic fungi play a key role in the digestion of ingested plant biomass in the rumen [17], where larger plant particles tend to be retained for longer than smaller plant particles. The selective ruminal retention of larger plant particles may be important in providing a more favourable ecological niche for the anaerobic fungi in terms the time taken to complete their life cycle, which according to Orpin [18] was estimated to take up to 16 h in the rumen. In support of this concept, it has been reported that animals fed diets containing chopped lucerne hay, with its longer retention time in the rumen, supported a larger population of ruminal anaerobic fungi compared to those fed a more rapidly digested diet of pelleted lucerne hay [19].

In the work of France et al. [20], fermentation gas production profiles were obtained from anaerobic fungal enrichment cultures growing on wheat straw particles of different sizes. Four particle sizes (approximating to 0.05, 0.46, 2.59, and 82.25 mm^2^) were tested and similar rates and extents of gas accumulation were obtained regardless of particle size. When the same range of particle sizes were subjected to fermentation by bacterial enrichment cultures, France et al. [20] observed that the rate and extent of fermentation was highly correlated to particle size; smaller particles, with a greater surface area, were degraded at a faster rate and to a greater extent than larger particles.

In the current study, we investigated the influence of cut-length of fresh and air-dried perennial ryegrass leaf-blades on the fermentation characteristics of an axenic culture of *Neocallimastix frontalis*. The leaf-blades cut-lengths were 0.5, 1.0, 2.0, and 4.0 cm, approximating surface areas of 50, 100, 200, and 400 mm^2^, respectively (assuming a 5 mm leaf width and that both the top and bottom surface of the leaf are the same area). To investigate the growth of *N. frontalis* on plant biomass that had not been subjected to physicochemical pre-treatment (autoclaving), unsterilized leaf-blades were used in these experiments. The research was undertaken to verify and extend the study published by France et al. [20]. The null hypothesis to be tested was that the cut-length of fresh and air-dried perennial ryegrass leaf-blades (within the range of 0.5–4.0 cm) does not affect the rate and extent of their digestion by *N. frontalis.* In addition, here we have adopted a novel approach to the gas production methodology, previously developed by Theodorou et al. [21,22], in an attempt to mitigate the lengthy lag-phase (often between 1 and 2 days) that is typical of anaerobic fungal growth in laboratory batch cultures.

## 2. Materials and Methods

### 2.1. Plant Material

Perennial ryegrass (*Lolium perenne*) AberDart (a diploid, intermediate-heading variety bred for improved fructan content), was grown from seed under controlled environment conditions (8 h 22 °C day/16 h 19 °C night; 800 µmol m^−2^ S^−1^ illuminations at 0.6 kilopascal) in a growth cabinet (Sanyo Gallenkamp PLC, Weiss Gallenkamp, Leicestershire, UK). The grass was watered three times a week and after 6 weeks of growth, leaf-blades were harvested by hand with scissors (blades were cut ca. 5 cm above soil level). The grass was then cut into 0.5, 1.0, 2.0, and 4.0 cm lengths and used immediately as a substrate for in vitro incubations, or was air-dried for subsequent use. Each leaf-blade, regardless of cut-length had two (top and bottom) cut ends. To dry in air, grass was spread evenly on the growth cabinet shelf where it was turned twice daily until a percentage dry matter of 85–90% was achieved. For co-ordination of experiments, grass grown for air-drying was sown and harvested 1 week in advance of the grass that was freshly harvested.

### 2.2. Maintenance and Pre-Growth of Neocallimastix frontalis Cultures

The monocentric anaerobic fungus *N. frontalis* R_E_1 [23] was kindly provided by Dr R. J. Wallace from stock cultures held within the Rowett Institute of Nutrition and Health at the University of Aberdeen (Aberdeen, UK). The culture was routinely grown at 39 °C in pre-warmed medium C [24] supplemented with 10% (*w/v*) barley straw [ground to pass through a 1 mm dry mesh screen using a Cyclotec 1093 Sample Mill (Foss, Warrington, UK)] as a carbon source and sub-cultured every 2 days.

For experimental work, 2 day-old *N. frontalis* cultures (as above) were inoculated (1 mL) into 160 mL serum bottles containing 100 mL of pre-warmed medium C supplemented with barley straw [2.5 g/L, i.e., 0.25% (*w/v*)]. Chloramphenicol (final concentration of 50 µg/mL) was added to the cultures, to prevent subsequent growth of plant epiphytic bacteria associated with the addition of the unsterilized leaf-blades. Cultures (100 mL) were grown on barley straw for approximately 2.5 days prior to addition of the cut leaf-blades. This time was chosen as it coincided with substrate (barley straw) limitation and the maximum zoospore titre of the cultures [25].

Visual inspection of all cultures, including those treated with chloramphenicol to inhibit bacterial growth, showed a lack of cloudiness (microbial swirl) which, together with the characteristic matts formed by the anaerobic fungi as they colonised plant biomass, provided evidence of the lack of microbial contamination in the growing cultures.

### 2.3. Modified In Vitro Gas Production Technique

**Figure 1:**
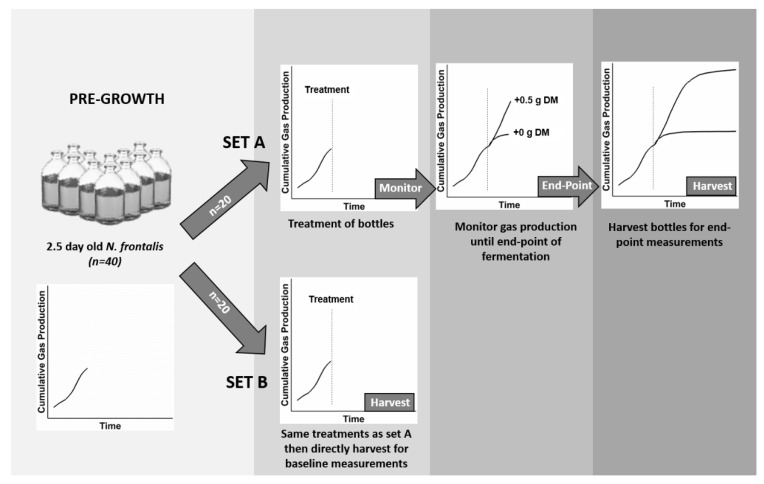
Schematic summary of the modified in vitro gas production technique. *Neocallimastix frontalis* cultures (*n* = 40) were first pre-grown for 2.5 days on 0.25% (*w/v*) barley straw before being divided into two sets of bottles (A and B). Both sets (*n* = 20) had the same five treatments (*n* = 4): either + 0.5 g dry matter (DM) of one of four different test substrates or + 0.0 g of DM in the case of the control bottles. Set B bottles were harvested at zero time for baseline measurements. Set A bottles were used to monitor the fermentation kinetics before being harvested for end-point measurements. Each replicated set (*n* = 4) of A bottles had a corresponding set (*n* = 4) of replicated B bottles.

In order to increase the zoospore titre (inoculum potential), and thereby rate of colonisation of cut leaf-blades, the gas production protocol for our work was altered from the standard protocol of Theodorou et al. [22]. Instead of adding cut leaf-blades to sterile media and inoculating with the fungus, leaf-blades were added to 2.5 day-old cultures of *N. frontalis* growing on 0.25% (*w/v*) barley straw. This modified protocol is represented diagrammatically in Figure 1. It was designed to permit use of a substantially larger (100 mL) pre-grown inoculum (which was substrate limited) to colonise and digest the newly added leaf-blades. The impact of barley straw on the subsequent leaf-blade fermentation analysis necessitated making gas production baseline measurements from bottles containing barley straw without leaf-blades as well as from bottles containing barley straw with leaf-blades.

The method of measuring gas production in growing cultures [21,22] was used to follow the fermentation of *N. frontalis,* both prior to and after the addition of fresh or air-dried cut leaf-blades (see below) to barley-straw containing cultures. The total volume of gas accumulated in the culture head-space was measured at regular time intervals using a pressure transducer. Following every reading, the head-space was vented, returning the pressure back to ambient conditions. The reading intervals were tailored to the amount of gas being produced, with more frequent readings taken during periods of rapid gas production.

### 2.4. In Vitro Fermentation of Different Cut-Lengths of Perennial Ryegrass Leaf-Blades by Cultures of N. frontalis Pre-Grown on Barley Straw

An experiment was conducted to investigate the ability of cultures of *N. frontalis* pre-grown on 0.25% (*w/v*) barley straw to ferment 4.0, 2.0, 1.0, and 0.5 cm cut-lengths of fresh perennial ryegrass leaf-blades. Forty bottles of *N. frontalis* pre-grown on barley straw were prepared in total. After their final reading of gas production on barley straw alone (54 h after initial inoculation), leaf-blades (approximately 0.5 g DM, accurately weighed) were added to 16 of these bottles (four bottles per leaf-blade cut-length); four additional, control bottles received no leaf-blades. These 20 bottles were referred to as set A. Leaf-blades were also added to 16 of the remaining 20 bottles, referred to as set B bottles with four additional bottles receiving no leaf-blades.

Leaf-blades were added to opened bottles under a stream of CO_2_. Control bottles not receiving leaf-blades were just opened, flushed with CO_2_, and closed. After additions, to set A bottles, they were gently agitated and returned to the incubator. Upon all additions, the gas pressures in each of the bottles were returned to ambient. Gas production in the set A bottles was then monitored using pressure transducer measurements as described above. After additions to set B bottles, the bottles were gently agitated and then destructively harvested for baseline measurements, enabling subsequent calculation of barley straw apparent DM loss, perennial ryegrass DM loss, and fermentation end-products produced from the perennial ryegrass (see Section 2.5). Once the gas production in the set A bottles had plateaued (at 168 h), these bottles were removed from the incubator and harvested in exactly the same manner as the set B bottles.

Bottles were destructively harvested as follows. Samples of culture supernatant were collected to measure the following main aqueous anaerobic fungal fermentation products: acetate, formate, and lactate. Particulate residues were recovered by vacuum-filtration through a weighed glass microfibre filter (Whatman^®^ 1.6 µm GF/A, Whatman International Limited, Maidstone, Kent, UK), with the bottle rinsed three times with approximately 100 mL of deionized water to ensure full recovery of all particulate residues. The filters with particulate residues were then freeze-dried to constant weight.

A further experiment was conducted to compare the effect of air-drying leaf-blades on their fermentation by *N. frontalis* using two different cut-lengths (i.e., fresh 0.5 cm, fresh 4.0 cm, air-dried 0.5 cm, and air-dried 4.0 cm). The 0.5 and 4.0 cm cut-lengths of fresh and air-dried leaf-blades were added 57 h after initial inoculation and subjected to the protocol described above using two replicate sets of bottles (A and B); within each set there were four bottles per treatment/control.

### 2.5. Determination of Perennial Ryegrass Fermentation End-Products, Initial Dry Matter Loss, and Apparent Dry Matter Loss

Acetate concentrations were determined in culture filtrates using gas chromatography as previously described [26]. Formate and total (D and L) lactate concentrations were determined enzymatically, using formate and lactate dehydrogenase methods, respectively, as previously described [16]. Metabolite production from perennial ryegrass was calculated by correcting for metabolite production from straw, using the mean value of the control bottles at the relevant harvest point (i.e., set A or B).

Initial perennial ryegrass DM loss was determined by freeze drying the residues recovered by filtration washing procedures from the set B bottles and taking into account the following: dry weight of plant material originally added to the bottle, glass microfibre filter paper weight, and the average of the particulate residue dry weight recovered from the barley straw control bottles. Apparent perennial ryegrass DM loss was determined in a similar manner using the particulate residues recovered from the set A bottles harvested at the end of the incubation period. Since monocentric anaerobic fungal biomass remaining on residues at the end of fermentation is minimal, due to zoospore release and autolysis [22], direct measurements of anaerobic fungal biomass were not undertaken.

### 2.6. Statistical Analysis

All statistical analyses were performed using GenStat [27]. For each bottle, gas volumes were regression corrected by fitting a linear regression model to volume and pressure readings. Cumulative volume was calculated, and the volume of gas at the end of the pre-growth phase was subtracted from the cumulative volume at each subsequent time point in the treatment phase. The mean profile (mL/g straw DM) was calculated for the control bottles (no grass addition) harvested at the final time point, and subtracted from each grass-treated profile to estimate the net cumulative grass profile from the perennial ryegrass, and then adjusted for dry weight of grass. The model of France et al. [20] was fitted in its linear form to the mean net accumulation data for each treatment. Estimates of the half-life (t_50_) and the fractional rate at t_50_ were derived as detailed by France et al. [20]. Model fitting was bootstrapped with a minimum of 500 repetitions. Median values for estimates of model parameters and derived quantities were determined and compared between treatments using the 2.5 and 97.5 percentiles as the 95% confidence intervals for comparison between treatments.

As indicated above, initial perennial ryegrass DM loss, apparent perennial ryegrass DM loss, and metabolite production from perennial ryegrass was calculated by correcting for residual straw DM and metabolite production from straw using the mean value of the control bottles at the relevant harvest point. Effects of treatment were examined by analysis of variance within harvest time either as a one-way design (leaf-blade cut-length) or a 2 × 2 factorial (leaf-blade cut-length × leaf-blade condition, i.e., fresh or air-dried). Where appropriate, treatment effects were partitioned using linear and nonlinear contrasts. The Student–Newman–Keuls test was used where comparison of more than two means was necessary.

## 3. Results

### 3.1. Effect of Cut-Length of Fresh Perennial Ryegrass Leaf-Blades on Their Fermentation by N. frontalis

**Figure 2:**
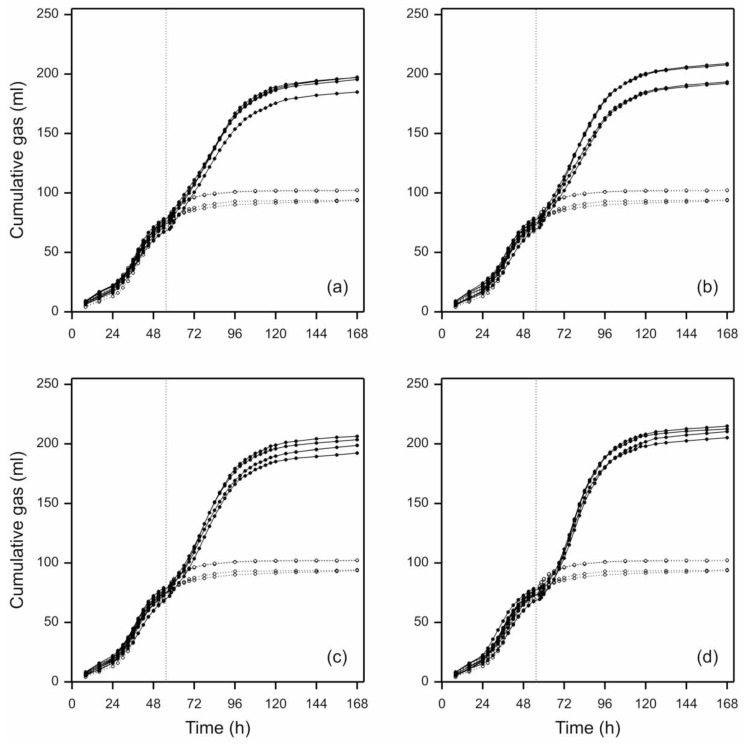
Individual total cumulative gas production profiles produced by *N. frontalis* cultures before and after addition of fresh perennial ryegrass of different cut-lengths: (**a**) 0.5 cm, (**b**) 1.0 cm, (**c**) 2.0 cm, (**d**) 4.0 cm; black lines. Dotted black lines with open symbols in each panel indicate cumulative gas production profiles for control bottles which had no leaf-blades added. The vertical grey dotted lines indicate the time of leaf-blade addition (at 54 h). Four individual bottles are plotted for each particle length, and three for the control (due to an outlier bottle being removed).

Prior to addition of perennial ryegrass leaf-blades, the mean value for the total amount of gas accumulated for all the pre-grown *N. frontalis* cultures (40 in total, i.e., set A and set B) was 79.7 mL g per 0.25 g straw added to each bottle (or 318.9 mL/g straw DM) with a coefficient of variation (c.v.) of 4.2%. This indicates that all the cultures behaved similarly prior to treatment and were appropriate for random treatment allocations as described above. The total cumulative gas production profiles of the individual profiles following treatment indicated that *N. frontalis* displayed minimal lag time and responded rapidly to the addition of new substrate, with the exception of the controls where no leaf-blades were added (Figure 2). Moreover, although comparisons of fitted profiles pre- and post- leaf-blade additions were not required for this research, the rate of gas production after leaf-blade addition was not dissimilar to that obtained on barley straw prior to leaf-blade addition (Figure 2). Figure 2 also demonstrates the exceptionally long lag time (approximately 25 h) that is typically observed with anaerobic fungal growth curves [22].

**Figure 3:**
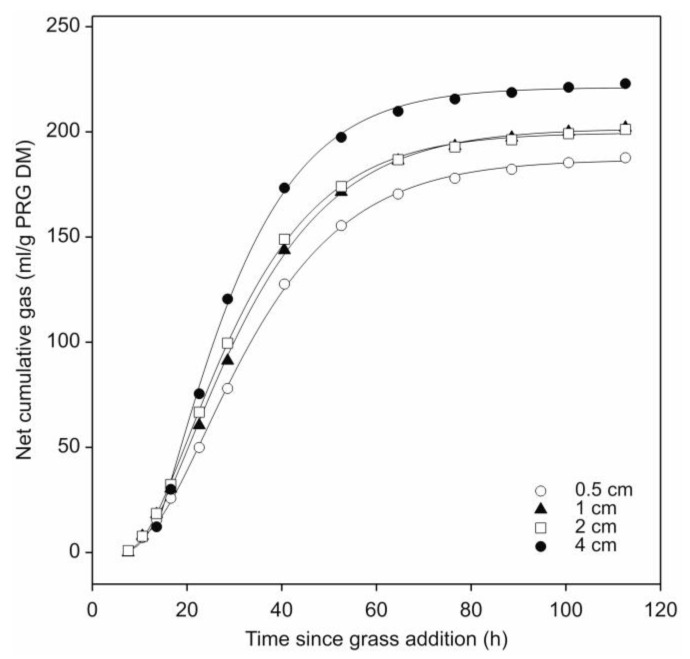
Effect of leaf-blade cut-length on cumulative gas production by *N. frontalis* fermentation of fresh perennial ryegrass (PRG). The plot is a representation of the fitted profiles generated using the model of France et al., [20]. Symbols represent mean net volumes for four replicated bottles with differing cut-lengths as identified in the figure. Time since grass addition is relative to the start of leaf-blade additions for each treatment.

Following subtraction of the barley straw gas production profiles and the curve fitting procedure, comparison of the fitted profiles for leaf-blades showed that gas production was significantly affected (*p* < 0.05) by cut-length (Figure 3; Table 1). The longest leaf-blades (4.0 cm) produced significantly more gas than the shortest (0.5 cm) (*p* < 0.05), with the 1 and 2 cm cut-lengths producing an intermediate amount that did not differ significantly (*p* > 0.05) from each other. In terms of the kinetics of gas production, the fractional rate of gas production also increased with leaf-blade cut-length but only the 4.0 cm length was found to differ significantly despite the gas production half-life significantly decreasing with each increase in leaf-blade length (*p* < 0.05) (Table 1).

Leaf-blade cut-length also had a significant but opposite effect on apparent DM loss and formate production (*p* < 0.05) (Table 2). Thus, while apparent DM loss decreased linearly (*p* = 0.003) as particle length increased, formate production increased linearly (*p* = 0.024) with increasing particle length, although significant differences were only observed between cultures supplied with 4.0 and 0.5 cm cut-lengths (data not shown). No significant effects were observed on initial DM loss or acetate and lactate production (*p* > 0.05) (Table 2).

### 3.2. Effect of Two Different Cut-Lengths of Fresh and Air-Dried Leaf-Blades of Perennial Ryegrass on Their Fermentation by N. frontalis

**Figure 4:**
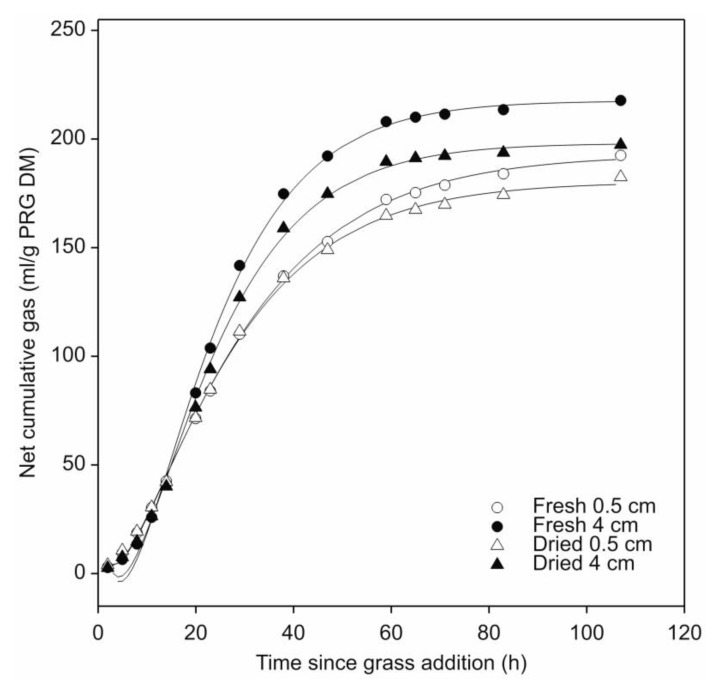
Effect of leaf-blade cut-length (0.5 or 4.0 cm) and forage condition (fresh or air-dried) on cumulative gas production by *N. frontalis* fermentation of perennial ryegrass (PRG). The plot is a representation of the fitted profiles generated using the model of France et al. [20]. Symbols represent mean net volumes for four replicated bottles of fresh or air-dried leaf-blades of differing cut-lengths as identified in the figure. Time since leaf-blade addition is relative to the start of leaf-blade additions for each treatment.

The effect of leaf-blade condition (fresh or air-dried) and cut-length (0.5 or 4.0 cm) on perennial ryegrass fermentation by *N. frontalis* was assessed. Prior to addition of perennial ryegrass leaf-blades, all the pre-grown barley straw cultures behaved similarly. The mean total amount of gas accumulated for all the pre-grown *N. frontalis* cultures (40 in total, i.e., set A and set B) was 60.1 mL per 0.25 g straw added to each bottle (or 240.3 mL/g straw DM) with a c.v. of 6.3%. As previously observed, the *N. frontalis* cultures responded rapidly and consistently to the perennial ryegrass leaf-blade addition (Figure S1). No effect of leaf-blade condition (fresh or air-dried) on gas production was observed, but a significant interaction (*p* < 0.05) between forage condition and leaf-blade cut-length occurred (Figure 4; Table 3). Gas production significantly differed (*p* < 0.05) within both the fresh and air-dried leaf-blades in terms of cut length. However, only gas production half-life values for cultures fermenting fresh perennial ryegrass were found to significantly differ (*p* < 0.05), with a longer half-life for the 0.5 cm compared to the 4.0 cm lengths. Similarly, the fractional rate values were only significantly different (*p* < 0.05) for cut-length in the cultures fermenting fresh perennial ryegrass, where cultures incubated with the 4.0 cm length had the greater rate.

Increased leaf-blade cut-length marginally but significantly decreased apparent DM loss (*p* < 0.001) and resulted in an increase in the production of acetate (*p* < 0.001) and formate (*p* = 0.023) (Table 4). Leaf-blade cut-length had no effect or interaction with forage condition (*p* > 0.05) (Table 4). Although the highest values for acetate and formate were obtained with cultures treated with the 4.0 cm cut-length, this treatment had the lowest values for apparent DM loss as had been previously observed (Table 2). Initial DM loss and lactate production were unaffected by leaf-blade cut-length, or air drying, or their interaction. 

## 4. Discussion

Before discussing the results obtained with leaf-blades, it is first necessary to provide a critique of the gas production methodology employed in this study. To our knowledge, the gas production methodology for determining anaerobic fungal growth kinetics in this study, of using a pre-grown culture to seed a second fermentation of newly added plant biomass, is novel and has not been used in previous anaerobic fungal research. In the current study, a lag time of approximately 24–36 h was recorded following the initial inoculation of barley straw (Figure 2 and Figure S1). Many studies have recorded similar or longer lag times (e.g., [28,29]) and this is reportedly due to the low zoospore titre in the initial fungal inoculum [22]. In the current work, it cannot be assumed that the barley straw cultures inoculated on different days (for experiments 1 and 2) were inoculated with the same number of zoospores. This is because *N. frontalis* is monocentric and zoospores alternate with vegetative thalli. Moreover, there is no easy method for quantifying zoospore titre prior to inoculation (e.g., such as optical density readings for bacterial inocula). Therefore, as the zoospore titres in the inoculum are unlikely to be exactly the same, cultures inoculated on different days are unlikely to have the same initial fermentation profiles (lag time and growth acceleration phase) although they will produce similar amounts of cumulative gas as this is determined by the organic matter content of the medium. Differences in inoculum potential represent an inherent problem when comparing the fermentation kinetics of anaerobic fungi in batch culture, as evidenced in the current work. Prior to perennial ryegrass leaf-blade addition, the same pre-growth protocol resulted in 318.9 mL/g straw DM in the first experiment and just 240.3 mL/g straw DM in the second experiment. A longer lag time and reduced acceleration phase in the second experiment resulted in different gas production profiles between the two experiments. A lower zoospore titre on inoculation in the second experiment is the most likely cause of this difference. Ultimately, however, given that the same amount and type of organic matter was present in the barley straw cultures, they all accumulated the same amount of gas (Figure 2 and Figure S1). 

The methodology deployed herein, by effectively increasing the size of the inoculum for the leaf-blade fermentations, successfully minimized the lag phase (Figure 3 and Figure 4). This observation is important from a biotechnological perspective. Many industrial fermentations employ similar methodology to reduce or indeed remove lag times and enhance productivity. Examples include anaerobic digestion where it takes many weeks post start-up to develop a productive methanogenic population. Once established, declines in productivity are avoided by addition of discrete quantities of feedstock while an equivalent proportion of the digestate is removed [30,31].

Two further key and novel aspects of the gas production methodology were as follows. (1) Growth of perennial ryegrass under ‘clean-room’ conditions and adding chloramphenicol to culture media to ensure that epiphytic bacteria on leaf-blade surfaces made a negligible contribution to the fungal fermentation of the forage. (2) Determining fermentation kinetics using fresh or dried plant material that had not been autoclaved prior to incubation with the fungus. To our knowledge, most in vitro studies where anaerobic fungi are cultured on plant biomass employ autoclaving to ensure culture medium sterility prior to inoculation. Fungal fermentation kinetics are, therefore, determined from plant biomass that has first been subjected to elevated temperature and pressure. Pre-processing of agricultural residues and green wastes is a key step in using them for fuel or in the biorefinery production of ethanol and other chemicals [32,33]. Pre-treatments using temperature and pressure tend to be effective in hydrolysing hemicellulose and releasing soluble sugars [34]. Although crystallinity can be reduced, cellulose polymers can remain almost unaltered [34]. The range of temperatures, pressures, and times used to bring about these biomass changes tend to be above those used in a conventional autoclave. However, autoclaving should be considered as a physical pre-treatment and although not acknowledged in anaerobic fungal research, it may affect the degradation characteristics of the plant biomass under study. Moreover, from the perspective of a biotechnological process, it is extremely important to demonstrate that costly (in terms of energy consumption) pre-treatments, such as autoclaving, are not required.

The anaerobic fungal fermentation of leaf-blades in this study yielded data that appears counter intuitive. Fermentation of fresh leaf-blades of 4.0 cm cut-length by *N. frontalis* produced 18.4% more gas, yet retained 11.2% more apparent DM relative to the 0.5 cm cut-length leaf-blades. Soluble fermentation products were similarly enhanced, in line with gas production. By summation of formate, acetate, and lactate (mmoL/g DM loss; Table 2) it can be calculated that the 4.0 cm leaf-blades produced 32% more fermentation end-products than the 0.5 cm leaf-blades, despite the fact that the shorter cut-length leaf-blades showed greater apparent DM loss.

Due to the nature of the *N. frontalis* life cycle, and the fact that the fungus requires complex media for optimal growth, it was not possible to quantify fungal growth and produce even a partial carbon-balance in these experiments. This would have enabled a more focused discussion on the underlying mechanism(s) associated with apparent DM loss and the production of fermentation end-products. The cell walls of *N. frontalis* contain chitin [18], which could be used as a biomarker for fungal growth [35,36]. However, chitin determination will not distinguish between living and dead fungus and its content in fungal biomass varies depending on the morphology of the fungus; anaerobic fungi are known to be highly pleomorphic [37,38,39]. Other markers of fungal biomass (e.g., specific fungal proteins) could be used. However, *N. frontalis* is a monocentric fungus with a determinate (finite) life span. Thus, once the fungus has sporulated, it no longer contains nuclear material and rapidly autolyses [40,41,42], making comparative biomass concentration measurements somewhat meaningless.

Fungi sporulate at substrate–water or substrate–air interphases to enable spore dispersal. This process of sporogenesis is common and well-documented for sexual and asexual reproduction processes in mycology. Spores of many saprophytic fungi are adapted to aerial dispersal, so much so that their spores are regarded as the most prevalent particles that are airborne [43]. Aquatic fungi in the division *Chytridiomycota*, which are also zoosporic fungi and closely related to the *Neocallimastigomycota*, typically produce zoosporangia and zoospores at substrate–water interphases [44]. The anaerobic fungal literature contains many descriptions of fungal sporangia liberating zoospores at cut and/or damaged plant particle surfaces [45,46]. It was observed that while sporulation was common at cut surfaces, it was seldom encountered at intact epidermal surfaces [45].

The methodology employed in this work exposed perennial ryegrass leaf-blades to significantly more zoospores than would have been encountered in conventional anaerobic fungal batch culture. As the life cycle of monocentric anaerobic fungi has been estimated to be anything between 8 and 26 h [40,47], the higher zoospore titre (inoculum potential) would have resulted in a high level of coordinated growth and almost synchronized sporulation during the leaf-blade incubations. We, therefore, hypothesise that the enhanced apparent DM loss of shorter cut-length leaf-blades observed in our work was related to a greater extent of sporulation, relative to that occurring with longer cut-length leaf-blades, due to the higher amount of cut ends per unit of plant biomass. *N. frontalis* growing on shorter cut-length leaf-blades will encounter cut surfaces more frequently than when growing on leaf-blades of longer cut-lengths and, therefore, sporulation at the cut surfaces of shorter leaf-blades will be more frequent per unit of plant biomass.

Due to the monocentric nature of the growth of *N. frontalis*, once it has sporulated, it will autolyse, giving rise to greater dry matter loss in cultures containing shorter cut-length leaf-blades than those containing the longer leaf-blades. The sporulation process also explains why more gaseous and aqueous fermentation end-products were produced from cultures growing on longer leaf-blades. With longer leaf-blades, as the fungus is less likely to encounter a cut surface it will, therefore, continue to grow vegetatively with the associated production of fermentation products. Further work, using more exacting analytical procedures and an anaerobic fungus that grows well on defined media (e.g., [29]) is now required to confirm this hypothesis.

In the second experiment, the effect of cut-length observed in the first experiment was confirmed. No direct effect of perennial ryegrass condition (i.e., fresh or air-dried) on its fermentation by *N. frontalis* was found. However, a significant interaction between perennial ryegrass cut-length and forage condition was observed. When taking into account cut-length, significantly more gas was produced from the fresh compared to air-dried leaf-blades (Table 3), although no corresponding effect on soluble fermentation products was observed (Table 4). The reason for this effect on gas production is not entirely clear. Air-dried leaf-blades are dehydrated and contain no viable plant cells. This is not the case for fresh leaf-blades as while plant cells at the cut ends of fresh plant material are no longer viable, the remainder are intact and progressively die [48]. Furthermore, during air-drying a proportion of the water soluble carbohydrate (WSC) content will have been converted to CO_2_ via respiration during the initial stages of the gentle drying process (under diurnal illumination) used in this study resulting in a decrease in WSC content. Thus, differences in the WSC content of fresh and air-dried leaf-blades may account for the production of less gas in air-dried leaf-blade fermentations. Further research, using the novel gas production approach presented in this study, to assess the impact of plant based factors on the fermentation of forage by anaerobic fungi is warranted, particularly because current understanding is primarily based on the use of material that has been autoclaved prior to fermentation. 

## 5. Conclusions

The anaerobic fungal fermentation of fresh and air-dried perennial ryegrass leaf-blades in this study yielded data that appears counter intuitive. In the first experiment, investigating different cut-lengths of fresh leaf- blades, fermentation of 4.0 cm cut-lengths produced 18.4% more gas and 32% more fermentation end-products, yet retained 11.2% more apparent DM relative to 0.5 cm cut-length leaf-blades. Results intermediate to the above values were obtained for the 1.0 and 2.0 cm cut-length leaf-blades. The second experiment, which investigated fresh and air-dried leaf-blades of 4.0 and 0.5 cm cut-lengths, fermentation of fresh leaf-blades produced results similar to those obtained in the first experiment. The 4.0 cm cut-lengths, relative to the 0.5 cm cut lengths, produced more (17.9%) gas and more (26.9%) fermentation end-products, yet retained 4.9% more apparent DM loss. However, although DM loss values were similar, the air-dried cut-lengths produces significantly less gas and fermentation end-products relative to their fresh cut-length counterparts. In conclusion, the null hypothesis, that cut-length of fresh and air-dried leaf-blades of perennial ryegrass does not affect their fermentation by *N. frontalis* has been disproved, and the alternative hypothesis, that leaf-blade cut-length does impact on the rate and extent of leaf-blade fermentation has been upheld. A possible explanation for this is that cut-length affects anaerobic fungal growth, due to the extent of sporulation being negatively influenced by increased leaf-blade length. Modifications of the existing gas production methodology employed in this study will be of value in similar studies, and highlights the potential for biotechnological exploitation of these unique microorganisms. In particular, the methodology enabled an increase in the size of the effective inoculum and the growth of *N. frontalis* to proceed on an un-autoclaved substrate with minimal lag time. The findings of this study, therefore, clearly highlight that lag-phase and forage cut-lengths are important factors that can be optimized when considering the biotechnological potential of anaerobic fungi.

## Figures and Tables

**Table 1:** Effect of fresh perennial ryegrass (PRG) leaf-blade cut-length on *N. frontalis* fitted total gas production (GP) data and derived parameters.

Parameter	Leaf-Blade Cut-Length (cm) *				*p*
	0.5	1.0	2.0	4.0	
Gas production (mL/g PRG DM ^#^)	186.7 ^a^ (184.1–188.8)	201.6 ^b^ (199.4–203.4)	199.6 ^b^ (197.1–201.3)	221.1 ^c^ (217.5–224.7)	<0.05
					
GP Half-life (t_50_) (h)	31.8 ^a^ (30.9–32.7)	30.1 ^b^ (29.3–30.8)	28.2 ^c^ (27.8–28.9)	26.4 ^d^ (24.6–27.6)	<0.05
					
Fractional rate of GP at t_50_ (h^−1^)	0.0450 ^a^ (0.0417–0.0479)	0.0462 ^a^ (0.0427–0.0489)	0.0494 ^a^ (0.0462–0.0516)	0.0568 ^b^ (0.0517–0.0646)	<0.05

* 95% confidence intervals are indicated in brackets and superscript letters indicate particle lengths which significantly differ (*p* < 0.05); ^#^ DM = dry matter.

**Table 2:** Effect of fresh perennial ryegrass (PRG) leaf-blade cut-length on initial and apparent dry matter (DM) loss, and production of aqueous fermentation end-products by *N. frontalis*.

Parameter	Leaf-Blade Cut-Length (cm)				s.e.m.	*p* *	*p* lin *	*p* dev *
	0.5	1.0	2.0	4.0				
Initial DM Loss (g/g PRG DM)	0.014	0.006	0.036	0.048	0.0175	0.332	0.102	0.832
Apparent DM loss (g/g PRG DM)	0.685	0.657	0.637	0.608	0.0147	0.019	0.003	0.576
Formate (mmoL/g Apparent DM loss)	2.30	3.30	2.89	4.00	0.408	0.068	0.024	0.311
Acetate (mmoL/g Apparent DM loss)	2.61	2.82	2.59	3.03	0.129	0.106	0.063	0.222
Lactate (mmoL/g Apparent DM loss)	1.27	1.39	1.25	1.10	0.082	0.165	0.061	0.437

* Significance of the effect of leaf-blade cut-length (*p*) is partitioned into linear (*p* lin) and nonlinear (*p* dev) contrasts.

**Table 3:** Effect of perennial ryegrass (PRG) condition (fresh or air-dried) on *N. frontalis* fitted total gas production (GP) data and derived parameters at two different leaf-blade cut-lengths.

Parameter	Fresh Leaf-Blades *		Air-Dried Leaf-Blades *		*p*
	0.5 cm	4.0 cm	0.5 cm	4.0 cm	
Gas production (mL/g PRG DM ^#^)	192.7 ^b^ (187.9–195.7)	227.2 ^c^ (223.4–233.6)	180.2 ^a^ (174.3–184.1)	198.0 ^b^ (194.9–204.7)	<0.05
					
GP half-life (t_50_) (h)	25.7 ^a^ (24.9–26.5)	23.5 ^b^ (22.7–24.6)	23.4 ^ab^ (23.1–25.1)	23.4 ^b^ (22.6–24.5)	<0.05
					
Fractional rate of GP at t_50_ (h^−1^)	0.0397 ^a^ (0.0371–0.0436)	0.0539 ^b^ (0.0471–0.0612)	0.0437 ^ab^ (0.0397–0.0494)	0.0526 ^b^ (0.0455–0.0592)	<0.05

* 95% confidence intervals are indicated in brackets and superscript letters indicate significantly different treatment means (*p* < 0.05); ^#^ DM = dry matter.

**Table 4:** Effect of perennial ryegrass (PRG) leaf-blade condition (fresh or air-dried) on initial and apparent dry matter (DM) loss, and production of aqueous fermentation end-products by *N. frontalis* at two different leaf-blade cut-lengths.

Variate	Length (L)	Condition (C)		Mean	C or L s.e.m. *	C × L s.e.m.	*p*		
		Fresh	Air-Dried				C	L	C × L
Initial DM loss	0.5 cm	0.054	0.026	0.040	0.092	0.0131	0.180	0.245	0.137
(g/g PRG DM)	4.0 cm	0.028	0.029	0.028					
	Mean	0.041	0.028						
									
Apparent DM loss	0.5 cm	0.662	0.655	0.658	0.0036	0.0050	0.895	< 0.001	0.281
(g/g PRG DM)	4.0 cm	0.629	0.634	0.631					
	Mean	0.645	0.644						
									
Formate	0.5 cm	2.79	3.41	3.10	0.204	0.288	0.179	0.023	0.476
(mmoL/g PRG DM loss)	4.0 cm	3.76	3.97	3.86					
	Mean	3.28	3.69						
									
Acetate	0.5 cm	2.50	2.65	2.57	0.108	0.153	0.882	< 0.001	0.280
(mmoL/g PRG DM loss)	4.0 cm	3.47	3.27	3.37					
	Mean	2.99	2.96						
									
Lactate	0.5 cm	2.26	2.08	2.17	0.072	0.102	0.054	0.644	0.714
(mmoL/g PRG DM loss)	4.0 cm	2.35	2.09	2.22					
	Mean	2.31	2.09						

* s.e.m., standard error of the mean.

## Supplementary Materials

The following are available online at https://www.mdpi.com/2076-2607/8/11/1774/s1, Figure S1: Individual total cumulative gas production profiles produced by *N. frontalis* cultures before and after addition of fresh or air-dried perennial ryegrass of different cut-lengths (a: 0.5 cm fresh, b: 4.0 cm fresh, c: 0.5 cm air-dried, d: 4.0 cm air-dried; black lines). Dotted black lines with open symbols in each panel indicate cumulative gas production profiles for control bottles which had no leaf-blades added. The vertical grey dotted lines indicate the time of leaf-blade addition (at 57 h). Four individual bottles are plotted for each cut-length, and for the control bottles.
