# Supplementary material for: Cut-Lengths of Perennial Ryegrass Leaf-Blades Influences In Vitro Fermentation by the Anaerobic Fungus Neocallimastix frontalis
Source: Microorganisms. 2020 Nov 11;8(11):1774. doi: 10.3390/microorganisms8111774 (PMC7696013; doi:10.3390/microorganisms8111774)

Supplementary Figure S1: Individual total cumulative gas production profiles produced by *N. frontalis* cultures before and after addition of fresh or air-dried perennial ryegrass of different cut-lengths (a: 0.5 cm fresh, b: 4.0 cm fresh, c: 0.5 cm air-dried, d: 4.0 cm air-dried; black lines). Dotted black lines with open symbols in each panel indicate cumulative gas production profiles for control bottles which had no leaf-blades added. The vertical grey dotted lines indicate the time of leaf-blade addition (at 57h). Four individual bottles are plotted for each cut-length, and for the control bottles.

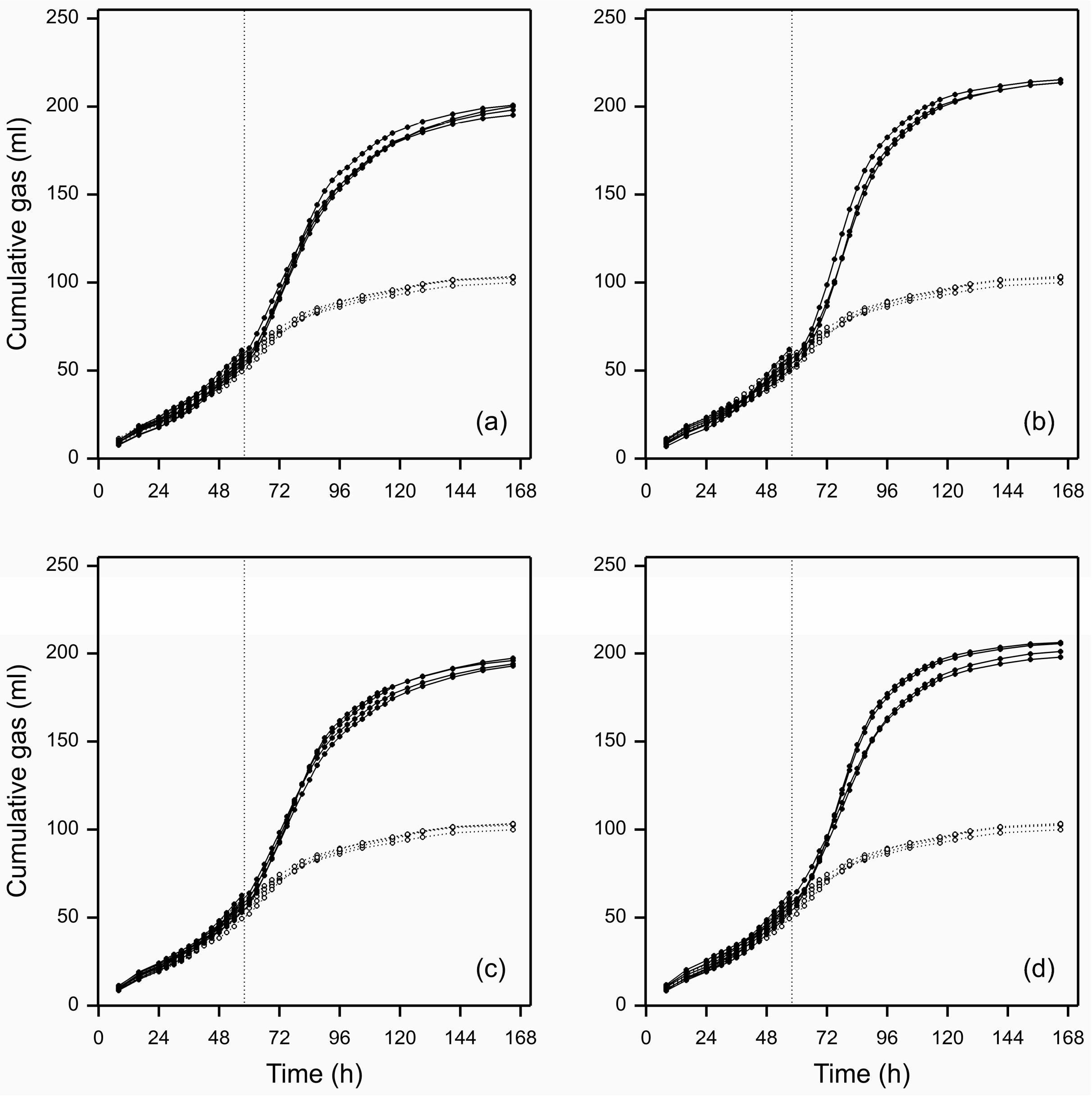

Supplement: Supplementary file 1 [file microorganisms-08-01774-s001.pdf]
